# Should Elective TIPS be Placed in Non‐Abstinent Patients With Alcohol‐Related Cirrhosis?

**DOI:** 10.1002/ueg2.70154

**Published:** 2025-11-29

**Authors:** Marika Rudler, Dominique Thabut

**Affiliations:** ^1^ Hepatology Department Sorbonne Université AP‐HP Pitié‐Salpêtrière Hospital Intensive Care Unit Paris France; ^2^ Brain Liver Pitié‐Salpêtrière (BLIPS) Paris France; ^3^ INSERM Centre de Recherche Saint‐Antoine (CRSA) Institute of Cardiometabolism and Nutrition (ICAN) Paris France

**Keywords:** alcohol, cirrhosis, tips

Transjugular intrahepatic portosystemic shunt (TIPS) has become the standard of care for patients with cirrhosis and severe decompensation, such as refractory/recurrent ascites, and for high‐risk patients with acute variceal bleeding (AVB) [1]. The European Association for the Study of the Liver (EASL) recently published clinical practice guidelines for TIPS, likely due to the growing number of patients who could benefit from this interventional technique [2]. Even with over 100 studies published annually on the subject, largely due to improvements in technique and a focus on better patient selection, whether controlling the underlying liver disease improves outcomes after TIPS placement remains an open question. A particularly relevant question is: in a world where alcohol‐related liver disease (ALD) is still a leading cause of cirrhosis, should we place a TIPS in patients who continue to drink alcohol?

In the current study by Schwartz et al. [3], the authors aimed to investigate the effect of alcohol use after TIPS placement on outcomes and survival. They conducted a retrospective study in two centers in Vienna from 2000 to 2022 and included 248 patients who received TIPS, primarily for ascites (70%). During a median follow‐up of 30 months, 63.7% of patients remained abstinent. There was no significant difference between non‐abstinent and abstinent patients in terms of further decompensation and hepatocellular carcinoma (HCC) (70.0% vs. 63.9%, *p* = 0.405 and 7.8% vs. 9.5%, *p* = 0.822, respectively). However, the incidence of acute‐on‐chronic liver failure (ACLF) (55.6% vs. 29.1%, *p* < 0.001), all‐cause mortality (72.2% vs. 34.8%, *p* = 0.009), and liver‐related death (53.3% vs. 34.8%, *p* = 0.007) were significantly higher in non‐abstinent patients. The authors also performed a subgroup analysis in non‐abstinent patients (*n* = 90) and compared outcomes to those from a cohort of patients with ALD who did not receive a TIPS (No TIPS patients, *n* = 50). Ascites and spontaneous bacterial peritonitis (SBP) were significantly more frequent in patients not treated with TIPS, but there were no significant differences in other outcomes, such as variceal bleeding, mortality, and ACLF. The authors concluded that careful patient selection is crucial for TIPS placement in ALD patients and that close follow‐up is mandatory after TIPS, suggesting support programs that focus on psychiatric assistance, physical activity, and social reintegration plans to facilitate alcohol abstinence.

Overall, the data analysis clearly illustrates countless hours of work, for which the authors are to be commended, and the expertise of the team is recognized worldwide. Yet, the harmful impact of heavy alcohol consumption and recurrence in patients with alcohol‐related cirrhosis is long‐established (and until very recently, was based on older studies), but the reader must fight against the initial impression that these results are obvious. Only 2 years ago, Louvet et al. [4] published a large prospective study conducted in a multicentric cohort of patients with compensated alcohol‐related cirrhosis, which clearly established the benefit of no alcohol consumption (< 1 glass/week) on survival and liver event‐free survival, and the dose‐dependent impact of alcohol over time on those two outcomes. Nevertheless, data on alcohol abstinence after TIPS are rarely reported, in elective [1], salvage [5], or preemptive settings [6]. From this perspective, the study by Schwartz et al. [3] is likely the first to provide such information, even if the reader should remember that abstinence was based on self‐declaration and highly nonspecific biological data (such as GGT or mean corpuscular volume). With the increasing use of specific biomarkers like phosphatidylethanol, it is well‐established that patients often underestimate their alcohol intake, especially those with a history of excessive alcohol consumption [7]. Looking at the results themselves, the absence of a difference between abstinent and non‐abstinent patients regarding further decompensation seems surprising when ACLF occurrence is significantly lower in abstinent patients (and for this specific outcome, Kaplan‐Meier curves intersect at 1 year). Lastly, regarding the comparison between non‐abstinent patients treated with or without TIPS, the reason why “no TIPS” patients were not treated with TIPS is not provided, which likely suggests a selection bias, making the interpretation of the results somehow questionable. Other methodological issues could be discussed, including the absence of data on abstinence at baseline, the exclusion of overt hepatic encephalopathy (HE) alone as a decompensating event, the exclusion of patients with a follow‐up of less than 3 months, and the exclusion of patients with hepatic venous pressure gradient < 16 mmHg in the “no TIPS” group.

The question of whether we should place a TIPS in a patient who is not abstinent (and perhaps wait for abstinence?) will not be answered by the current study. Recent data suggest that, in the specific setting of concomitant severe alcohol‐related hepatitis and acute variceal bleeding, the benefit of preemptive TIPS placement is highly probable in terms of survival and further decompensation, without increasing the risk of overt HE [8]. We hope that prospective multicentric studies on elective TIPS will begin soon, using accurate questionnaires for alcohol consumption and specific biomarkers such as phosphatidylethanol to evaluate the prognostic impact of alcohol recurrence. This could be quantified, for instance, in “glass‐years” in the same way as “pack‐years” for tobacco, and using a time‐dependent covariate. We need data to assist clinicians in decision‐making, and should not forget that in clinical practice, the vast majority of our patients with alcohol‐related cirrhosis are not abstinent [9], and they likely do not benefit from standard‐of‐care treatment due to the stigmatizing effect of non‐abstinence.

## Funding

The authors have nothing to report.

## Conflicts of Interest

MR: speaker for Gore, Abbvie. DT: speaker for Gore, Abbvie, Gilead, consulting Alfasigm, Genfit.

## Data Availability

Research data are not shared.
